# Impact of Anti-amyloid-β Monoclonal Antibodies on the Pathology and Clinical Profile of Alzheimer’s Disease: A Focus on Aducanumab and Lecanemab

**DOI:** 10.3389/fnagi.2022.870517

**Published:** 2022-04-12

**Authors:** Mingchao Shi, Fengna Chu, Feiqi Zhu, Jie Zhu

**Affiliations:** ^1^Department of Neurology, Neuroscience Center, The First Hospital of Jilin University, Changchun, China; ^2^Cognitive Impairment Ward of Neurology Department, The Third Affiliated Hospital of Shenzhen University Medical College, Shenzhen, China; ^3^Division of Neurogeriatrcs, Department of Neurobiology, Care Sciences and Society, Karolinska Institute, Karolinska University Hospital Solna, Stockholm, Sweden

**Keywords:** Alzheimer’s disease, amyloid-β, monoclonal antibodies, lecanemab, aducanumab, treatment

## Abstract

Alzheimer’s disease (AD) is the most prevalent form of age-related dementia in the world, and its main pathological features consist of amyloid-β (Aβ) plaque deposits and neurofibrillary tangles formed by hyperphosphorylated tau protein. So far, only a few AD treatments approved have been applied in the clinic, but the effects of these drugs are limited only for partial symptomatic relief to patients with AD and are unable to alter AD progression. Later, all efforts for AD treatments with targeting the pathogenic factors were unsuccessful over the past decades, which suggested that the pathogenesis of AD is complex. Recently, disease-modifying therapies (DMTs) that can change the underlying pathophysiology of AD, with anti-Aβ monoclonal antibodies (mabs) (e.g., aducanumab, bapineuzumab, gantenerumab, solanezumab, and lecanemab) have been developed successively and conducted in clinical trials based on the theory that a systemic failure of cell-mediated Aβ clearance contributes to AD occurrence and progression. In the review, we summarized recent studies on the therapeutic effects and clinical trial results of these mabs in patients with AD. Specifically, we focused on the discussion of the impact of aducanumab and lecanemab on AD pathology and clinical profiles. The review provides a possible evidence for applying immunotherapy with anti-Aβ mabs in AD and analyzes lessons learned from these clinical trials in order to further study the therapeutic and adverse effects of these anti-Aβ mabs on AD.

## Introduction

Alzheimer’s disease (AD) as a chronic neurodegenerative disorder is the most common age-associated dementia accompanied by progressive loss of memory and cognitive functions as well as synaptic dysfunction (Alexiou et al., 2020). By 2050, there will be 115 million people, of which 10–30% of population aged 65 years or above are affected by AD. It becomes a public health predicament in the world, and there is a significant impact on the direct cost of AD to the society (Povova et al., 2012; Masters et al., 2015; Angelucci et al., 2019).

The two main neuropathological hallmarks of AD are amyloid-β (Aβ) plaques and neurofibrillary tangles formed by intracellular accumulation of hyperphosphorylated tau protein (Serrano-Pozo et al., 2011; Bloom, 2014; Uddin et al., 2020a). One of them, the amyloidogenic pathway, may be involved in the pathogenesis of AD. Aβ peptides, such as Aβ_40_, Aβ_42_, and Aβ_43_, are the products of the successive cleavage of amyloid precursor protein (APP) by β- and γ-secretases, and they can assemble into insoluble beta-sheet fibrillar aggregates that deposit extracellularly in the brain parenchyma and cerebral vasculature, causing the damage of synaptic structure and function, and neuronal atrophy in the hippocampus area and then spreading to cortical regions, resulting in cognitive impairment and dementia (Lu et al., 2013; Khan et al., 2014). In addition, inflammation can also contribute to the development of AD (Guillot-Sestier and Town, 2013; Goetzl et al., 2018; Webers et al., 2020). So far, only a few drugs approved have been applied in the clinic for treatment in AD, such as the acetylcholinesterase inhibitors and the non-competitive *N*-methyl-D-aspartate receptor antagonist. However, these drugs are unable to alter AD progression, only for partial symptomatic relief (Olivares et al., 2012). Based on the amyloid cascade hypothesis, it is believed that the clearance of brain Aβ plaques may treat AD and cease disease progression, which promoted the development of innovative anti-Aβ drugs to prevent Aβ aggregation in the brain in the past 30 years. Unfortunately, all efforts in the treatment of AD targeting the pathogenic Aβ or tau have failed in the past, which proposed that the pathogenesis of AD is more complex and multifactorial (Zhu et al., 2020). At present, anti-Aβ therapies are still under debate.

There is a growing evidence that innate immune plays an important role in AD’s etiology, although the central nervous system (CNS) has long been considered an immune-privileged site (Guillot-Sestier and Town, 2013; Webers et al., 2020). Furthermore, the new insights on the pathogenesis and therapy of AD also indicated the adaptive immune response contributing to the deposition of Aβ in the brain and misfolded tau proteins (Ashraf et al., 2018; Uddin et al., 2020b), which might open new perspectives in the treatments for AD with active and passive anti-Aβ immunotherapies clearing brain Aβ deposits (Ciccocioppo et al., 2020). Recently, anti-Aβ monoclonal antibodies (mabs) as the immune therapeutic approaches have been investigated as a treatment for AD, including aducanumab, bapineuzumab, gantenerumab, solanezumab, and lecanemab. These mabs are distinct in selectivity for polymorphic variants and recognize epitopes based on the specific portion and conformations of Aβ (Arndt et al., 2018). Among these mabs, aducanumab and gantenerumab partially target oligomers, while most mabs clear insoluble Aβ plaques (Tolar et al., 2020a). Thus, these mabs target Aβ *via* the distinct metabolic pathways to remove Aβ and soluble misfolded oligomeric antecedents or to prevent the adoption of misfolded conformations of Aβ, declining the levels and toxicity of Aβ in the brain (Olzscha et al., 2011; Guo and Lee, 2014). All these mabs can reduce the levels of Aβ peptides, 1–40 and 1–42 in cerebrospinal fluid (CSF), or plasma at various degrees with different doses, but the effects of the mabs on p181-tau level differed, which have been discussed in the below sections.

In the review, we discussed the effects of mabs, including aducanumab, bapineuzumab, gantenerumab, solanezumab, and lecanemab on AD, especially the concentration on the impacts of aducanumab and lecanemab on AD pathology and clinical manifestations. These mabs have been tested in participants with early AD, preclinical stage of familial AD, and asymptomatic participants with a high risk of developing AD. To date, the outcomes of clinical trials seem to stand by the amyloid hypothesis in the pathogenesis of AD. However, there were a series of clinical trial failures with applying these mabs, which is still a question on further development of Aβ-targeting drugs. To provide further evidence applying immunotherapy for disease-modifying therapies (DMTs) in AD and analyze lessons learned from previous and current clinical trials, we summarized the updated studies on these mabs in clinical trials in patients with AD and further evaluated the possibility and effectiveness of the immunotherapies with mabs in AD.

## Immunotherapies With Mabs in Patients With Ad and Its Animal Models

The immunotherapeutic approaches are promoting Aβ clearance from the brain of AD *via* injection of Aβ antigens (active immunization) or anti-Aβ antibodies (passive immunization; Panza et al., 2012). Passive immunization with anti-Aβ antibodies can enhance Aβ clearance from plasma and the CNS, leading to a decline in Aβ burden through the peripheral sink mechanism of action (Zhang and Lee, 2011; Imbimbo et al., 2012). After intravenous (IV) administration by these anti-Aβ mabs, they bind to soluble Aβ peptides in the periphery and sequester them into an immune complex that can be removed from the circulation, thereby reducing plasma Aβ levels. To keep the balance between Aβ oligomers (ABOs), aggregates, and plaques in the CNS, amyloid can transform to produce soluble monomers and can pass through the blood–brain barrier (BBB) to restore the decreased plasma Aβ levels. Finally, the low CNS Aβ level reduced Aβ-related cellular toxicity and pathology (Zhang and Lee, 2011).

Recently, treatment with the second generation of anti-Aβ mabs in AD has made a great progress in clinical trials. A crucial feature shared by these mabs is their ability to engage neurotoxic soluble ABOs, albeit to various degrees. Until now, the results from phase III clinical trials with most mabs were unsuccessful. However, aducanumab in its phase III study obtained relative positive outcomes, despite the controversy (Panza et al., 2019), which supports to continue the testing of the anti-Aβ mabs in the treatment of AD. Currently, in this study, some of the mentioned mabs to decline brain Aβ levels are still probing in clinical trials (Decourt et al., 2021). The therapeutic and side effects of the mabs in AD and its animal models are presented in Table 1.

**TABLE 1:** Treatments of patients with AD and animal models with anti-Aβ monoclonal antibodies.

Pre-clinical and clinical studies	Anti-Aβ monoclonal antibodies	Immune response and pathological changes after therapies	Clinical profiles changes after therapies	Side effects	References
Tg2576 transgenic mice Prodromal or mild AD patients	Aducanumab	Binding parenchymal Aβ, and soluble and insoluble Aβ↓(mice) Amyloid plaque at week 54 ↓by PET scan (patients)	Slowing clinical progression via detections by MMSE and CDR-SB; No changes on NTB or FCSRT (patients)	ARIA-E and superficial siderosis (patients) (Sevigny et al., 2016) ARIA-E 35.2%, ARIA-E was highest in ApoE4 + subjects (Swanson et al., 2021)	Sevigny et al., 2016; Swanson et al., 2021
Mild to moderate AD	Bapineuzumab	No significant changes in whole-brain volume.	No significant improvement in clinical symptoms (ADAS-Cog/11, DAD, NTB, Z-score, CDR-SOB and Dependence Scale)	ARIA-E and cerebral microhemorrhage ↑	Vandenberghe et al., 2016
Prodromal AD patients	Gantenerumab (105 or 225 mg/4 weeks)	Biomarkers of neural and synaptic degeneration↓; PET SUVr↓ in 225 mg dose group; MRI volumetry: No difference; CSF: t-tau and p-tau↓	No significant improvement in clinical symptoms in 105 or 225 mg (CDR-SB, ADAS-Cog 13, MMSE, and FAQ, FCSRT with immediate recall total recall, CANTAB, and NPI-Q)	ARIA-E 6.6% (105 mg), 3.5% (225 mg). ARIA-H 22.9% (105 mg), 16.2% (225 mg)	Ostrowitzki et al., 2017
Prodromal to moderate AD	Gantenerumab (1200 mg/4 weeks)	PET: resulted in robust Aβ plaque removal at 2 years	Slowed clinical decline with higher Aβ removal in 1,200 mg group (CDR-SB, ADAS-Cog 11, and MMSE)		Klein et al., 2019
DIAD mutation dominantly inherited Alzheimer’s disease (Salloway et al., 2021)	Gantenerumab (1200 mg/4 weeks) (Salloway et al., 2021)	PiB-PET: brain Aβ deposition ↓; CSF: Aβ42↑, t-tau and p-tau 181 ↓, NfL↑ slowed at year 4. 18F-FDG-PET and volumetric MRI: no difference in brain cortical metabolism or atrophy	No difference in cognitive decline between the gantenerumab and control	ARIA-E 19.2%	Salloway et al., 2021
Early Alzheimer’s disease	Lecanemab (10 mg/kg biweekly)	CSF: Aβ42↑, p-tau ↓ PET SUVr:brain Aβ↓at 18 months	Improvement in clinical symptoms (ADCOMS, ADAS-Cog14, CDR-SB) Greater reductions of cognitive decline in ApoE4 + subjects	ARIA-E 10% ARIA-H: 10.7% ARIA-E and ARIA-H was higher in ApoE4 + subjects Infusion reactions 19.9%	Swanson et al., 2021
Mild AD (Farlow et al., 2012; Honig et al., 2018)	Solanezumab	Total Aβ40 and Aβ42 in CSF↑ Unbound Aβ40 in CSF↓ Unbound Aβ42 in CSF↑ (Farlow et al., 2012; Honig et al., 2018)	No significant improvement (ADAS-cog14, MMSE, ADCS-iADL, FAQ, CDR-SB score) (Farlow et al., 2012; Honig et al., 2018)		Farlow et al., 2012; Honig et al., 2018
DIAD mutation dominantly inherited Alzheimer’s disease		CSF NfL ↑, Aβ PET, t-tau or p-tau181: No difference Brain cortical metabolism 18F-FDG-PET or atrophy: No difference	Faster cognitive decline in the solanezumab group vs. the control groups	ARIA-E was lower in solanezumab group	Salloway et al., 2021

### Aducanumab

Aducanumab, a human immunoglobulin 1 (IgG1) mab, can selectively target aggregated Aβ, including neuritic Aβ plaques and high molecular weight ABOs but excludes Aβ monomers (Sevigny et al., 2016; Linse et al., 2020). In the CNS, aducanumab displays a preference for parenchymal over vascular Aβ (Sevigny et al., 2016).

Treatments with anti-Aβ mabs in AD animal models have been very successful, which can improve cognitive functions and decrease brain pathology *via* microglial stimulation and prevention of Aβ aggregation (Novakovic et al., 2013; Vander Zanden and Chi, 2020). Aducanumab has been shown to have a clear therapeutic effect and effectively remove Aβ from the brain of mice. Aducanumab can enter the brain of transgenic (tg) mice and bind parenchymal Aβ and decline soluble and insoluble Aβ (Sevigny et al., 2016). Ten-month-old tgAPPPS1-21 mice (AD animal model) were treated chronically with aducanumab for 4 months of weekly dosing (10 mg/kg), which showed that aducanumab was obviously suppressed Aβ toxicity and enhanced phagocytosis and cell viability (Bastrup et al., 2021). To increase the brain levels of aducanumab, treatment with aducanumab combination together with ultrasound in APP23 mice, an AD model, can restore cognition in APP23 mice due to ultrasound scan with intravenously injected microbubbles, which temporarily opens the BBB, facilitating aducanumab entry into the brain (Leinenga et al., 2021). In Tg2576 mice, as an AD model, aducanumab lowered Aβ plaque in a dose-dependent manner in 9-month-old mice but not in 22-month old mice (Kastanenka et al., 2016; Sevigny et al., 2016), indicating that aducanumab was more effective in preventing Aβ aggregation than in clearing the existing amyloid plaques (Kastanenka et al., 2016). However, the cognitive or behavioral improvement did not occur after treatment with aducanumab in such mice (Kastanenka et al., 2016).

Aducanumab was approved as the first DMT for AD by the United States Food and Drug Administration (FDA) in June of 2021 (Dhillon, 2021). Aducanumab treatment was initiated in patients with mild cognitive impairment (MCI) or mild dementia stage of disease, and successful development of aducanumab is considered a milestone in the treatment of AD (Dhillon, 2021).

Aducanumab binds to Aβ plaques and ABO and stimulates microglia to clear Aβ by reducing brain Aβ in a dose- and time-dependent manner by slowing down cognitive impairment in prodromal or mild AD measured by Clinical Dementia Rating-Sum of Boxes (CDR-SB) and Mini-Mental State Examination (MMSE) scores (Sevigny et al., 2016; Linse et al., 2020). In a double-blind randomized and placebo-controlled conditions, phase Ib study, treatment with aducanumab, was obtained successful outcomes and showed to be a benefit to AD-associated MCI or mild AD dementia. Thus, aducanumab was advanced to phase III clinical trials in September 2015 (ClinicalTrials.gov, 2020a,b).

A study that recruited 196 patients with AD treated with aducanumab showed a similar result that aducanumab reduced Aβ plaques and slowly declined in clinical measures in patients with prodromal or mild AD (Budd Haeberlein et al., 2017). In addition, aducanumab revealed a significant efficacy on both clinical and biomarker outcomes (Haeberlein et al., 2020) and an acceptable safety and tolerability profile, as well as linear pharmacokinetics at a dose of ≤30 mg/kg in a single-dose study (Ferrero et al., 2016). Amyloid-related imaging abnormalities (ARIAs), the side effect associated with the removal of Aβ, were dose-dependent in the aducanumab-treated group. ARIAs occurred more often in ε4 allele of apolipoprotein E gene (APOE4) carriers, the strongest genetic risk factor for the late-onset AD, than non-carriers (Cummings et al., 2018; Lin et al., 2018), which was a main safety finding and could justify further application of aducanumab for the treatment of AD (Sevigny et al., 2016). Recently, the significant results demonstrated the modest but greatest efficacy in a phase III trial by aducanumab, supplying important Aβ validation as a therapeutic target (Schneider, 2020). Aducanumab has been demonstrated as a worthwhile application probably in dose− and treatment duration−related lowering of Aβ plaques across the phase Ib (PRIME trial), IV, and III studies (Schneider, 2020). The trial results were not always consistent and were accompanied by differences in the studies of 945 patients (Engage) and 803 patients (Emerge) in 2018, which displayed that aducanumab treatments were trending positive in the Emerge group and trending negative in the Engage group (Lin et al., 2018). The inconsistent results may be attributed to aducanumab entering the brain at low concentrations or lack of selectivity for the soluble ABOs (Knopman et al., 2021). Fortunately, several additional trials conducted in the Engage and Emerge groups later showed that patients in the Engage group treated with aducanumab experienced a slow decline similar to the Emerge group change in CDR-SB relative to the placebo group (ClinicalTrials.gov, 2020a,b). The trials showed excellent dose-dependent amyloid clearance in both groups, while inconsistency was observed in cognitive outcomes in these studies. Thus, the correct application of aducanumab dosage was essential for reducing clinical decline, brain Aβ, and CSF phosphorylated-tau levels in AD (Wang et al., 2016).

Herring et al. (2021) predicted the long-term clinical benefits of patients with early AD treated by aducanumab evaluated by a Markov modeling approach. The results were that aducanumab treatment caused 0.65 increased patient quality-adjusted life-years (QALYs) and 0.09 fewer nursing staff QALYs lost compared with patients treated with standard of care (SOC) (Herring et al., 2021). Therefore, the clinical trials with aducanumab are now still undergoing in the two large-scale phase III trials and will obtain the final results in 2023. The effect of aducanumab compared with other mabs on AD is described in the “Bapineuzumab, crenezumab, gantenerumab, and solanezumab” section.

From June 2021, aducanumab was approved to treat mild AD until today, which has been caused considerable medical and scientific controversy (Tampi et al., 2021). Although aducanumab does reduce Aβ, there is a lack of reliable evidence that it has significant benefits to patients with AD (Fleck, 2021). In phase III, among patients treated with high-dose aducanumab, ∼35% of patients occurred ARIA-related cerebral edema (ARIA-E), and ∼18–22.7% of patients had ARIA-related microhemorrhages (ARIA-H) or other side effects, such as headache, dizziness, and nausea. Most ARIA-E events occurred in the early stages of aducanumab treatment. These results were consistent with other clinical studies of anti-Aβ antibodies, and the risk of ARIA-E was reduced during subsequent treatment. These findings caused the scientists to confuse by the FDA’s decision, which was based on the reduction of a surrogate marker (Aβ) rather than on data showing clinical efficacy (Nisticò and Borg, 2021). Therefore, FDA calls for further evaluation of the effect of aducanumab on AD in 2021 (Kuller and Lopez, 2021). In February 2022, an article published in Neurology summarized all trials conducted and indicated that aducanumab significantly reduced Aβ plaques in the brain. However, it has not yet been proved whether it has an effect on AD-related symptoms. It is reported that about 40% of patients treated with aducanumab experienced brain swelling and bleeding as their side effects, but most side effects disappeared when aducanumab was stopped. So far, aducanumab is only approved for MCI and early AD but not for patients with moderate to severe AD. FDA recommends close monitoring with magnetic resonance imaging (MRI) in patients treated with aducanumab, and more in-depth studies are needed on many aspects of aducanumab treatment (Day et al., 2022).

Overall, aducanumab is the first approved mab for DMTs in mild AD, and the therapeutic effects obtained in clinical trials were inconclusive so far, which is required to conduct more clinic trials, especially in asymptomatic and Aβ-positive individuals. A phase IIIb open-label trial including 2,400 participants treated with aducanumab at 10 mg/kg/month injections for 2 years has been conducted, and safety and tolerability parameters are the primary endpoints, hoping to get definite results by the end of 2023 (Chiao et al., 2019; Knopman et al., 2021).

### Bapineuzumab, Crenezumab, Gantenerumab, and Solanezumab

The therapeutic effects of these exogenous mabs on AD are through targeting and removing brain Aβ, such as aducanumab targeting both ABOs and plaques, crenezumab targeting ABOs, gantenerumab targeting Aβ fibrils, and solanezumab targeting Aβ monomers. When compared with other mabs, bapineuzumab showed stronger immunoreactivity on fixed tissue samples than with sodium dodecyl sulfate-denatured samples on Western blots, indicating conformational preferences of this antibody (Zampar et al., 2020).

In a total of 17 clinical studies, 12,585 patients with AD were divided into several subgroups treated with aducanumab, bapineuzumab, crenezumab, gantenerumab, and solanezumab, respectively. The results revealed that aducanumab improved cognitive function by small effect sizes and declined Aβ detected by positron emission tomography (PET) and CSF p181-tau by large effect sizes. Solanezumab improved cognitive function by small effect sizes and enhanced CSF Aβ1-40 levels by a moderate effect size. Bapineuzumab, crenezumab, and gantenerumab had no effect on the improvement of clinical outcomes. Bapineuzumab and gantenerumab reduced CSF p181-tau by small and large effect sizes, respectively. Aducanumab, bapineuzumab, crenezumab, and gantenerumab increased ARIAs risk. The outcomes of all mabs pooled together showed that these mabs alleviated clinical symptoms by small effect sizes, caused biomarker improvements by large effect sizes, and enhanced ARIAs by a large effect size. In short, aducanumab exerts the most beneficial effects, followed by solanezumab (Avgerinos et al., 2021).

In addition, several clinical trials in patients with AD have also been conducted with aducanumab, bapineuzumab, gantenerumab, and solanezumab previously. In the randomized-controlled trials, the majority of the outcomes from 13 phase III trials using aducanumab, bapineuzumab, crenezumab, gantenerumab, and solanezumab were positive, and from 3 phase II trials using crenezumab and aducanumab were largely negative. As a significant adverse effect in the treatment groups, ARIAs were ranged between 0.2 and 22% (Loureiro et al., 2020). It was also observed that the therapeutic effect of bapineuzumab on AD showed a vague result in improving cognition accompanied by obvious side effects, such as vasogenic edema and rarely brain microhemorrhages (Panza et al., 2012). Gantenerumab displayed significant biomarker effects without clinical efficacy (Klein et al., 2019). Solanezumab, a humanized anti-Aβ mab directed against Aβ peptide neutralizing soluble Aβ, was reported to have a good safety profile during the phase II trial (Farlow et al., 2012). However, Honig et al. (2018) found that the treatment of mild AD with solanezumab at a dose of 400 mg for 1 month did not significantly affect cognitive decline.

Furthermore, quantifying the effects of aducanumab, bapineuzumab, gantenerumab, and solanezumab on oligomer production and aggregation kinetics, and correlating these effects with the affinity and stoichiometry of each mab for monomeric and fibrous Aβ showed that only aducanumab dramatically reduced the flux of ABOs (Linse et al., 2020). To prevent bapineuzumab treatment from microhemorrhages and vasogenic edemas in patients with AD, the single-chain variable fragments (scFvs) derived from bapineuzumab, which targets N-terminal of the Aβ peptide and recognizes monomers, oligomers, and fibrils, were used to treat 3xTg-AD mice, an animal model for AD. The results showed that 3xTg-AD mice partially recovered the values in brain volume, compared with the controls (Güell-Bosch et al., 2020). The therapeutic effect of scFvs was manifested as clearance of intracellular Aβ, reduction of neuronal loss, and improvement of cognitive impairment, and the treatment was safe (Esquerda-Canals et al., 2019b). In addition, scFvs more easily passed BBB and were co-localized with Aβ peptide in glia at the late phase post-injection, resulting in declining Aβ peptide levels in the brain (Esquerda-Canals et al., 2019a).

**Crenezumab,** as a humanized IgG4 mab, can bind to multiple forms of aggregated Aβ, including oligomers, fibrils, and plaques, to clear excess Aβ (Salloway et al., 2018), particularly it has a 10-fold higher affinity toward soluble oligomers that are primary drivers of Aβ-related neurotoxicity (Cummings et al., 2018). Thus, crenezumab can strongly inhibit oligomer-induced neurotoxicity (Lin et al., 2018) and block Aβ aggregation and promote Aβ disaggregation of oligomers (Ultsch et al., 2016). Unlike IgG1, crenezumab declines the activation of Fc-gamma receptors (Fcγ6Rs) on CNS macrophages preventing neuroinflammation caused by inflammatory cytokines and other inflammatory mediators that trigger Aβ neurotoxicity (Ultsch et al., 2016). Crenezumab can prevent from vascular side effects caused by IgG1 mab, such as ARIA-E, ARIA-H, and complement-dependent cytotoxicity. The safety of crenezumab treatment is obviously enhanced due to a lower risk of inducing ARIAs (Crehan and Lemere, 2016; Graham et al., 2017).

In two phase I studies including healthy participants, it was proved that crenezumab was well-tolerated in healthy participants with an acceptable safety profile (Dolton et al., 2021). However, phase 3 clinical trials recruited 750 patients with prodromal to mild AD, and the outcomes were completely negative, since there was no difference between crenezumab and placebo subgroups or within the prodromal vs. mild AD subgroups assessed by several parameters, such as Alzheimer’s Disease Assessment Scale–Cognitive Subscale score (ADAS-Cog) and MMSE (Salloway et al., 2018), suggesting that crenezumab had no therapeutic effect on AD symptoms. Although there were about 94% of participants with at least one adverse event, most adverse events were mild or moderate in a completed phase Ib study with crenezumab. Moreover, participants showed a very low percentage of new ARIA-E and ARIA-H, and the safety of crenezumab treatment was acceptable (Guthrie et al., 2020). In addition, the experience gained from two unfinished phase II clinical trials in patients with very mild AD was to test high-dose crenezumab in such patients in the future (Cummings et al., 2018), and the view has also been supported by a phase Ib study (Yoshida et al., 2020). Recently, a phase II clinical trial for crenezumab that recruited patients in the preclinical phase of AD, who carried the presenilin 1 E280A autosomal dominant mutation, was conducted and completed in February 2022.

In the AD animal models, intracerebral injection by crenezumab in Tg2576 mice did not show any inflammatory response, indicating that crenezumab can significantly inhibit inflammation in the brain (Ultsch et al., 2016), which is beneficial to AD.

**Gantenerumab,** as a fully human anti-Aβ IgG1 mab, targets Aβ fibrils with subnanomolar affinity (Klein et al., 2021) and binds at a conformational epitope with N-terminal and central amino acids in a configuration that cannot be achieved with the structure of Aβ monomers (Bohrmann et al., 2012). Particularly, gantenerumab is suitable for long-term DMT for patients with AD.

The part 1 of phase II/III study for the evaluation of the efficacy and safety of gantenerumab at different doses in 799 patients with prodromal AD was conducted through measuring changes in the CDR-SB score, brain Aβ levels, cognition, and behavior, as well as through other tests for a total of 26 months. Since it is impossible to receive the efficacy on the primary and secondary endpoints in the clinic trial after a 26-month period, it had to early terminate in 2017 (Ostrowitzki et al., 2017). The *post hoc* analysis of gantenerumab’s data showed a little bit positive result in patients with faster progressors. Therefore, the clinical trial regimes have been altered according to several factors, such as injecting doses, observed periods, and the amount/different stages of recruited patients in order to evaluate the efficacy and safety of gantenerumab. Unfortunately, the results were also failed (Salloway et al., 2021). However, the trials that enrolled 81 patients treated with gantenerumab at 225 mg dose showed that Aβ levels in one-third of the participants declined below the threshold for Aβ positivity at the end of the treatment (Klein et al., 2018). In patients with prodromal to moderate AD, gantenerumab treatment up to 1,200 mg once every month showed obvious Aβ removal (Klein et al., 2019) and continued to decline Aβ plaque at 3 years after the start of the treatment (Klein et al., 2021).

In addition, a phase II study was performed to assess the safety, tolerability, and biomarker efficacy of gantenerumab vs. solanezumab in patients with a risk of the rare autosomal dominant AD (ADAD) gene mutation in 2012. The results from the study were negative. Gantenerumab and solanezumab at low doses could not significantly slow cognitive decline and were not better than placebo after treatment for 48 months (Farlow et al., 2020). Although gantenerumab was proved ineffective in another study, dose-dependent effects observed in clinical and biomarker endpoints suggested that testing with higher dosing for long term may be necessary to achieve clinical efficacy (Ostrowitzki et al., 2017).

Gantenerumab and other two mabs, solanezumab and crenezumab, were tested in 144 carriers of ADAD for the evaluation of their efficacy in two long-term preventive studies (Dominantly Inherited Alzheimer Network Trials Unit Adaptive Prevention Trial [DIAN-TU-APT] and Alzheimer Preventive Initiative-ADAD) for 4 years. The outcomes of both studies were also negative, indicating that these mabs could not be prevented from cognitive decline in ADAD that may not be triggered by Aβ (Imbimbo et al., 2021). A similar result was obtained in another study with dominantly inherited AD (DIAD) received by gantenerumab, solanezumab, and placebo, respectively, for 4–7 years. Finally, the result demonstrated that both gantenerumab and solanezumab had no beneficial effect on cognitive measures compared with controls. The asymptomatic subjects did not display cognitive decline; symptomatic participants had declined before reaching the target doses (Salloway et al., 2021). Although gantenerumab significantly lowed Aβ plaques, CSF total tau, and p181-tau, as well as weakened the enhancement of neurofilament light (NfL), it had no effect on the improvement of cognition. Furthermore, ARIAs-E was found by 19.2, 2.5, and 0% in gantenerumab, placebo, and solanezumab groups, respectively (Salloway et al., 2021). Currently, an investigation of potential clinical benefits related to gantenerumab-induced Aβ-lowering in patients with prodromal-to-mild AD is ongoing as GRADUATE phase III trials (Klein et al., 2021), and it will be completed in November 2023.

In brief, gantenerumab is able to remove cerebral Aβ plaques and normalize Aβ42, tau, and p181-tau levels in CSF and inhibit NfL; therefore, it is necessary to further continue investigating gantenerumab at higher dosage in prodromal AD or AD by determining the effects of gantenerumab on the prevention and treatment of AD.

**Solanezumab,** as a humanized version of a murine antibody, is similar in its binding to crenezumab (Tian Hui Kwan et al., 2020). Solanezumab can reduce brain Aβ burden by altering CNS and plasma Aβ clearance in both tg mice of AD and patients through target engagement of solanezumab with soluble CNS Aβ peptides, which may lead to Aβ efflux into the periphery or disturbance of the fibrillar-soluble Aβ equilibrium that ultimately reduced soluble brain Aβ (DeMattos et al., 2001; Legleiter et al., 2004; Doody et al., 2014).

Previously, two clinical tri0061ls investigating solanezumab have been completed, which provided sufficient evidence that solanezumab is benefit to prodromal AD (Honig et al., 2018). In addition, solanezumab led to insignificant therapeutic benefits at the earlier stages of AD. Salloway et al. (2021) reported that a greater cognitive drop was observed in the solanezumab-treated group and did not show benefits to downstream biomarkers. Unimaginably, solanezumab obviously accelerated cognitive drop in both asymptomatic and symptomatic participants, and the failure further challenges the Aβ pathogenicity hypothesis in AD (Imbimbo et al., 2021). The reason for the failure of solanezumab treatment may be that its biological effect of removing brain Aβ plaques was not enough to cause the improvement of patients’ cognition. However, the incidence of ARIAs was 0.9 and 0.4% for solanezumab and placebo, respectively, indicating that solanezumab did not induce ARIAs displaying its great tolerability and safety (Doody et al., 2014).

Currently, to evaluate whether IV infusion of solanezumab can slow the rate of progression of cognitive decline and improves disease-related biomarkers in DIAD, a phase II/III randomized, double-blind, placebo-controlled study that recruited 490 participants is ongoing (DIAN-TU trial), which may determine the tolerability, toxicity, and adequate dose of solanezumab in the AD population and is planned to be completed in July 2022 (Decourt et al., 2021).

In conclusion, solanezumab therapy did not decrease cognition decline, but showed to reduce brain Aβ level. These findings provide moderate support for the continuous investigation of its effectiveness and safety.

### Lecanemab

Lecanemab is a humanized IgG1 of the mouse mab158 and can selectively bind to large, soluble Aβ protofibrils that are the most neurotoxic and contribute to the pathogenesis of AD (Logovinsky et al., 2016). It has been evidenced that lecanemab can reduce the pathogenic Aβ, prevent Aβ deposition, and selectively reduce Aβ protofibrils in the brain and CSF of AD animal models (Söllvander et al., 2015; Tucker et al., 2015).

Brain samples from Down syndrome (DS) that caused by trisomy of chromosome 21 leading to develop Aβ brain pathology followed by cognitive and behavioral deterioration, AD, and non-demented controls (NDCs) were analyzed different Aβ species by immunohistochemical staining with anti-Aβ antibodies. It was observed higher immunohistochemical staining of Aβ deposits with lecanemab in DS and AD compared with NDC, suggesting that lecanemab may be possible to retain DS’s cognitive abilities (Johannesson et al., 2021).

Previously, the safety and tolerability were investigated in patients with mild to moderate AD in the first clinical study with lecanemab. The results found that incidence of ARIA-E/H (E for edema, H for hemorrhage) assessed by MRI was comparable with that of placebo, and lecanemab was well-tolerated across all doses (Logovinsky et al., 2016). Modest efficacy has been observed in the highest doses of lecanemab and the risk of vasogenic edema limited higher dosing of lecanemab application, particularly in APOE4 carriers (Abushakra et al., 2016, 2017). In a randomized double-blind clinical trial, 609 subjects with early AD, MCI, and mild AD dementia were treated by lecanemab, and 245 subjects were treated by placebo. The results showed that lecanemab (10 mg/kg biweekly) significantly decreased brain Aβ, which was different when compared with the placebo group at 72 weeks, indicating in favor of active treatment with lecanemab (Swanson et al., 2020, 2021). The therapeutic effect of lecanemab was supported by changes in CSF biomarkers, and lecanemab was well-tolerated with 9.9% incidence of ARIAs-E at 10 mg/kg biweekly (Swanson et al., 2021). Lecanemab also showed a significant efficacy on both clinical and biomarker outcomes (Logovinsky et al., 2016). In the phases I and II (2b) trials, the outcomes suggested that lecanemab completely removed Aβ plaques from brain, alleviated cognitive decline, and had a low incidence ARIA-E in early AD (Wang et al., 2016; Swanson et al., 2021). As such, lecanemab may have a potential effect on AD pathology to slow down the progression of AD. Based on favorable preclinical findings and multiple clinical trial results, lecanemab is a potential viable mab’s drug for the treatment of AD.

For the evaluation of the efficacy of lecanemab on cognition in early AD compared with placebo, the phase III randomized, placebo-controlled, double-blind, parallel-group trial has been conducted. In the trials, lecanemab at 10 mg/kg has been administered intravenously once every 2 weeks, which results have not yet been published (Tian Hui Kwan et al., 2020; Swanson et al., 2021). Another study aims to investigate the efficacy and safety of lecanemab in preclinical AD, including the participants with either a first-degree relative diagnosed with dementia onset before age 75 with at least one APOE4 allele or high Aβ levels in brain or CSF, by a designed therapeutic protocol (5 mg/kg every 2 weeks for 2 months, then 10 mg/kg every 2 weeks for 2 years, and 10 mg/kg every 4 weeks for 4.5 years), which may provide clinical evidence to determine its efficacy and safety for applying lecanemab in such patients (Swanson et al., 2018; Tian Hui Kwan et al., 2020).

Lecanemab seems to be the most promising treatment for AD among these mabs due to the decline of brain Aβ levels, the alleviation of cognitive decline, and a low incidence ARIA-E. It has a moderate therapeutic effect and better safety. However, the results from several clinical trials were largely negative and failed to show clinically relevant effects in patients with clinically manifest or prodromal dementia. It is necessary to conduct further investigations on the efficacy and safety of lecanemab.

## Discussion

Amyloid hypothesis is considered to be related to the etiology of AD, but nearly all pharmaceutical therapies targeting Aβ have failed in the clinic during the past about 20 years, indicating that the pathogenesis of AD is quite complex and should be multifactorial. Despite many problems regarding immunotherapy for AD and these knowledge gaps in the pathogenesis of AD, the studies have still progressed in developing more anti-Aβ mabs for the treatment of AD.

Currently, several anti-Aβ mabs, such as aducanumab, bapineuzumab, gantenerumab, solanezumab, and lecanemab, have developed and conducted in clinical trials. However, the results of most clinical trials with these mabs were largely negative, which raised many questions about the future development of AD drugs and has proven challenging. The main problems of treatments with these mabs are failing to show clinically relevant effects in patients with clinically manifest or prodromal dementia, and the high incidence of ARIAs caused by some mabs, indicating that the risk of adverse events outweighs the benefits of the treatments. Thereby, majority of clinic trials with these anti-Aβ mabs therapies did not meet primary endpoints and stopped the clinical trials. To conduct further studies and analyze lessons learned from these trials, several questions should be addressed before these trials can be used as evidence to support the Aβ cascade hypothesis of AD and to treat such patients.

First, it is crucial to further explore the role of amyloid hypothesis in the pathogenesis of AD. Although it has been believed that Aβ42 is the major Aβ toxic species linked with AD pathogenesis, recent studies proposed that Aβ40 is also involved in the AD development and progression *via* a more complex mechanism (Strozyk et al., 2003; Fagan et al., 2006; Blennow et al., 2010; Michno et al., 2019). Hence, future directions merit in exploring the role of Aβ40 in the pathogenesis of AD. Simultaneously, it is a key point to study the relationship between cerebral Aβ levels and cognitive decline. Later, it is warranted to develop the agents, such as immunotherapies with mabs effectively inhibiting ABO formation and their toxic effect, as well as to conduct their clinical trials. In addition, the synergistic benefits regarding the combinations of anti-inflammation, anti-Aβ, and anti-tau drugs merit further study due to multifactor participation in the etiology of AD.

Second, the benefits of treatment with mabs in preclinical AD, MCI due to AD, and mild AD must be further provided sufficient evidence and confirmed by the continuation of randomized, placebo-controlled, double-blind clinical trials to determine the effect of anti-Aβ mabs therapy in asymptomatic carriers of autosomal-dominant mutations related to early-onset AD (Loureiro et al., 2020). Previously, the late-stage trials in AD treated with the mabs targeting distinct species of Aβ had provided relatively favorable evidence that inhibited soluble ABOs only, not Aβ plaque by aducanumab, lecanemab, gantenerumab, and donanemab, which may be an effective approach to improve the clinic, slow, or stop AD progression. Another powerful evidence was due to higher levels of ABOs in APOE4 carriers’ brains, and the efficacy of anti-Aβ mabs was greater, which strongly supports for continuing clinical trials with these mabs, especially, further conducting clinical trials with aducanumab and lecanemab. It is noteworthy to choose the appropriate subjects with biomarker evidence and optimal dosage of mabs in the future clinical trials. It is necessary to choose DIAD as the appropriate subjects, since they can be observed for AD-related biological changes decades before the onset of AD. Early intervention with mabs in the asymptomatic and symptomatic stages can delay or slow the progression of AD, because the pathologic process of AD begins decades prior to functional decline and diagnosis, which have confirmed by neuroimaging, biomarker, and clinical data studies (Bateman et al., 2012; Fleisher et al., 2012; Mielke et al., 2012). However, the timing of this previous intervention had been too late to impact on neurodegenerative process of AD (Callaway, 2012; Miller, 2012). Although aducanumab (Sevigny et al., 2016; Linse et al., 2020), crenezumab (Salloway et al., 2018), gantenerumab (Ostrowitzki et al., 2017), and solanezumab (Salloway et al., 2021) have been conducted in several clinical trials with the asymptomatic stages of the subjects, the outcomes were negative or vague, which may be due to short observation time and use of inappropriate doses. There is still a lack of reliable evidence and trials for the use of these mabs to prevent AD. It is crucial to conduct an early intervention with mabs, and the current ongoing phase III trials will hopefully give light to this critical issue. The optimal mab dosage is key to the success of clinical trials, because increasing dosage in the later period of AD might have a negative impact on the trial results (Salloway et al., 2021).

Third, it should be considered that ideal therapeutic drugs, such as mabs, should cross BBB efficiently and sustain the brain levels to prevent oligomer formation and inhibit their toxicity continuously (Tolar et al., 2020b) or alter the route of administration so that the drugs (mabs) can directly enter the brain *via* nasal route or intracerebral injection. AD process starts with amyloid buildup, while cognitive impairment is the last event during the pathological process (Jack and Holtzman, 2013); hence, DMTs with mabs must be performed early. Otherwise, treatment will be ineffective due to late treatment just like the current treatment situation (van Dyck, 2018).

## Conclusion

Recently, the novel highly specific mabs targeting Aβ as DMTs for AD have been developed and conducted in several clinical trials. Although most results from the clinical trials were unsuccessful, the mabs as the new generation of DMTs may offer an additional possibility of therapeutic options. These mabs have been proven to be relatively safe in humans and some of them were proved to have mild to moderate effects on declining brain Aβ levels and improving cognitive impairment. Among them, aducanumab and lecanemab have relatively good effects. However, the efficacy of these mabs in patients was uncertain, and there are still many questions to be solved. Future DMTs for AD should focus on preventing cognitive decline in cognitively unimpaired individuals with evidence of cerebral amyloidosis.

## Author Contributions

MS, FC, and FZ prepared the manuscript. FC and JZ helped to conceived and reviewed the manuscript. JZ conceived, wrote, and finalized the manuscript. All authors read and approved the final manuscript.

## Conflict of Interest

The authors declare that the research was conducted in the absence of any commercial or financial relationships that could be construed as a potential conflict of interest.

## Publisher’s Note

All claims expressed in this article are solely those of the authors and do not necessarily represent those of their affiliated organizations, or those of the publisher, the editors and the reviewers. Any product that may be evaluated in this article, or claim that may be made by its manufacturer, is not guaranteed or endorsed by the publisher.

## Abbreviations

AD: Alzheimer’s disease
A β: amyloid β
CNS: central nervous system
mabs: monoclonal antibodies
DMTs: disease-modifying therapies
IV: intravenous
BBB: blood–brain barrier
IgG1: immunoglobulin 1
mab: monoclonal antibody
ABO: A β oligomers
FDA: Food and Drug Administration
MCI: mild cognitive impairment
CDRS: Clinical Dementia Rating-Sum
MMSE: Mini-Mental State Examination
ARIAs: amyloid-related imaging abnormalities
APOE4: ε 4 allele of apolipoprotein E gene
QALYs: quality-adjusted life-years
SOC: standard of care
CSF: cerebrospinal fluid
ARIA-E: Alzheimer’s related imaging abnormality-cerebral edema
ARIA-H: Alzheimer’s related imaging abnormality microhemorrhages
tg: transgenic
PET: positron emission computed tomography
scFvs: single-chain variable fragments
Fc γ 6Rs: Fc-gamma receptors
ADAS-Cog: Alzheimer’s Disease assessment scale–cognitive subscale score
CDR-SB: clinical dementia rating –sum of boxes
ADAD: autosomal dominant AD
DIAD: dominantly inherited AD
NfL: neurofilament light
DS: Down syndrome
NDC: non-demented controls
MRI: magnetic resonance imaging
ADCS-ADL: Alzheimer’s Disease Cooperative Study Activities of Daily Living Inventory
CDRSB: Clinical Dementia Rating Sum of Boxes
CDR-SOB: Clinical Dementia Rating–Sum of Boxes
DAD: Disability Assessment for Dementia
FAQ: Functional Activities Questionnaire
FCSRT: Free and Cued Selective Reminding Test
NPI-Q: Neuropsychiatric Inventory Questionnaire
NTB: Neuropsychological Test Battery
PiB PET: Pittsburgh compound B positron emission tomography
p-tau: Phosphorylated-tau 181
SUVr: Standardized uptake value ratio
t-tau: Total tau
18F-FDG PET: Positron emission tomography with 2-deoxy-2-[fluorine-18]fluoro-D-glucose.
